# Supplementary material for: Impact of climate change on the distribution of insectivorous bats: Implications for small-scale farming in southern Mexico
Source: PLoS One. 2024 Dec 2;19(12):e0310623. doi: 10.1371/journal.pone.0310623 (PMC11611147; doi:10.1371/journal.pone.0310623)
Supplement: S1 File — (DOCX) [file pone.0310623.s001.docx]

4. With which ethnic group do you identify?

| 1 | Indigenous |  |
| --- | --- | --- |
| 2 | Afrodescendent |  |
| 3 | Mestizo |  |

**A. Personal information**

1. Gender:

Female Male Hombre

2. How many people live with you at home?

| 1 | 2 | 3 | 4 | 5 | 6 | 7 | 8 | 9 | 10 | 11 |
| --- | --- | --- | --- | --- | --- | --- | --- | --- | --- | --- |
|  |  |  |  |  |  |  |  |  |  |  |

3. Which is your main occupation?

| 1 | Farmer |  |
| --- | --- | --- |
| 2 | Merchandizer/Trader |  |
| 3 | Housewife |  |
| 4 | Other |  |

**B.** **Bat information**

8. In recent years, have you seen fewer or more bats in the area?

Yes No I don’t know

9. Why do you think the number of bats varies?

Yes No I don’t know

5. Have you seen bats in your field during the last year?

Yes No I don’t know

6. What do these bats eat?

7. Inside the fields, specifically in the maize, have you seen bats?

What do you think they do around the fields?

Yes No I don’t know

**D.** **Economic information**

14. How much money do you spend in pesticides?

**C. Field information**

10. Is the field of your property?

Yes No

11. Which is your average yield in a year?

12. Which is the extent of your field?

13. Is your maize production only for selfconsumption?

**E.Pests**

15. Imagine a scenario in which you had to spend more money

because you can’t control the pests in your field, would you be able to buy or

increase the amount of chemical pest control you buy?

16. If your answer is yes, what would you have to do to obtain the money?

What would you do if you can’t access extra money?

17. What would you do if you had to stop growing maize?
