# Supplementary material for: Impact of climate change on the distribution of insectivorous bats: Implications for small-scale farming in southern Mexico
Source: PLoS One. 2024 Dec 2;19(12):e0310623. doi: 10.1371/journal.pone.0310623 (PMC11611147; doi:10.1371/journal.pone.0310623)
Supplement: Attachment — Submitted filename: PONE_D_24_03606_.pdf [file pone.0310623.s004.pdf]

PLOS ONE

Impact of Climate Change on Insectivorous Bats and Implications for Small-Scale Farming in southern Mexico

--Manuscript Draft--

**Manuscript Number:**	PONE-D-24-03606
**Article Type:**	Research Article
**Full Title:**	Impact of Climate Change on Insectivorous Bats and Implications for Small-Scale Farming in southern Mexico
**Short Title:**	Climate change on insectivorous bats and small-scale farming
**Corresponding Author:**	Carolina Ureta<br>Universidad Nacional Autónoma de México Instituto de Ciencias de la Atmósfera y Cambio Climático: Universidad Nacional Autonoma de Mexico Instituto de Ciencias de la Atmosfera y Cambio Climatico<br>Mexico City, MEXICO
**Keywords:**	Bat-mediated pest control; Maize; climate change; economic value; Chiapas
**Abstract:**	Insectivorous bats preying on insect pests in croplands provide an important ecological service for agriculture and food security. This role is particularly relevant for small-scale farmers in Chiapas, southern Mexico, that rely on maize for subsistence. The impact of climate change is resulting in a decrease in insectivorous bat populations and an increase in insect pests. This study develops a four-step protocol to assess potential impact of climate change on insectivorous bats controlling insect pest of crops, and implications on small-scale farmers' livelihoods. We (1) used available official information to quantify costs of chemical pest control on the entire area under maize cultivation, (2) interviewed small-scale farmers to determine their perception on insectivorous bats as beneficial for preying on insect pests, (3) estimated the economic value of ecosystem services provided by insectivorous bats, and (4) projected impacts of climate change on future distributions of insectivorous bats in Chiapas. The estimated value of insect pest control by insectivorous bats that official statistics yield in Chiapas was $7.9 USD/ha for 2023. Conversely, the estimated costs resulting from interviews with small-scale farmers was $40.1 USD/ha for 2023. Most interviewed small-scale famers perceive a decline in bat population but are unaware that insectivorous bats consume large proportions of insect pests, and even fear the presence of bats in their crops. Projections of shifts in bat population due to climate change suggest potential declines of insectivorous bats in areas where small-scale farmers cultivate maize for subsistence. This scenario implied higher costs associated with chemical pest control, which in some instances may provoke that farmers abandon their crops. We highlight the complex interplay between ecosystem service (insectivorous bats controlling insect pests), social perception of role of bats in their crops, socioeconomic factors, and small-scale agricultural sustainability.
**Order of Authors:**	Carolina Ureta
	Mercedes Ramírez-Barrón
	Melanie Kolb
	Giovanna Gasparello
	Felipe Ruán-Soto
	Adán L. Martínez-Cruz
	Víctor Sánchez-Cordero
**Additional Information:**	
**Question**	**Response**
**Financial Disclosure**<br><br>Enter a financial disclosure statement that	Yes

Powered by Editorial Manager® and ProduXion Manager® from Aries Systems Corporation

describes the sources of funding for the work included in this submission. Review the submission guidelines for detailed requirements. View published research articles from *PLOS ONE* for specific examples.<br><br>This statement is required for submission and **will appear in the published article** if the submission is accepted. Please make sure it is accurate.<br><br>**Funded studies**<br>Enter a statement with the following details:<br>• Initials of the authors who received each award<br>• Grant numbers awarded to each author<br>• The full name of each funder<br>• URL of each funder website<br>• Did the sponsors or funders play any role in the study design, data collection and analysis, decision to publish, or preparation of the manuscript?<br><br>Did you receive funding for this work?	
Please add funding details.<br>as follow-up to "**Financial Disclosure**<br><br>Enter a financial disclosure statement that describes the sources of funding for the work included in this submission. Review the submission guidelines for detailed requirements. View published research articles from *PLOS ONE* for specific examples.<br><br>This statement is required for submission and **will appear in the published article** if the submission is accepted. Please make sure it is accurate.	Graduate scholarship from Conahcyt

Powered by Editorial Manager® and ProduXion Manager® from Aries Systems Corporation

**Funded studies**<br>Enter a statement with the following details:<br>• Initials of the authors who received each award<br>• Grant numbers awarded to each author<br>• The full name of each funder<br>• URL of each funder website<br>• Did the sponsors or funders play any role in the study design, data collection and analysis, decision to publish, or preparation of the manuscript?<br><br>Did you receive funding for this work?"	
Please select the country of your main research funder (please select carefully as in some cases this is used in fee calculation).<br>as follow-up to "**Financial Disclosure**<br><br>Enter a financial disclosure statement that describes the sources of funding for the work included in this submission. Review the submission guidelines for detailed requirements. View published research articles from *PLOS ONE* for specific examples.<br><br>This statement is required for submission and **will appear in the published article** if the submission is accepted. Please make sure it is accurate.<br><br>**Funded studies**<br>Enter a statement with the following details:<br>• Initials of the authors who received each award<br>• Grant numbers awarded to each author<br>• The full name of each funder<br>• URL of each funder website<br>• Did the sponsors or funders play any role in the study design, data collection and analysis, decision to publish, or preparation of the manuscript?<br><br>Did you receive funding for this work?"	MEXICO - MX
**Competing Interests**	No authos have competing interests

Use the instructions below to enter a competing interest statement for this submission. On behalf of all authors, disclose any competing interests that could be perceived to bias this work—acknowledging all financial support and any other relevant financial or non-financial competing interests.<br><br>This statement is required for submission and **will appear in the published article** if the submission is accepted. Please make sure it is accurate and that any funding sources listed in your Funding Information later in the submission form are also declared in your Financial Disclosure statement.<br><br>View published research articles from *PLOS ONE* for specific examples.<br><br>**NO authors have competing interests**<br><br>Enter: *The authors have declared that no competing interests exist.*<br><br>**Authors with competing interests**<br><br>Enter competing interest details beginning with this statement:<br><br>*I have read the journal's policy and the authors of this manuscript have the following competing interests: [insert competing interests here]*<br><br>* typeset	
**Ethics Statement**<br><br>Enter an ethics statement for this submission. This statement is required if the study involved:<br><br>• Human participants<br>• Human specimens or tissue<br>• Vertebrate animals or cephalopods<br>• Vertebrate embryos or tissues	N/A

• Field research<br><br>Write "N/A" if the submission does not require an ethics statement.<br><br>General guidance is provided below. Consult the submission guidelines for detailed instructions. **Make sure that all information entered here is included in the Methods section of the manuscript.**	

Powered by Editorial Manager® and ProduXion Manager® from Aries Systems Corporation

**Format for specific study types**<br><br>**Human Subject Research (involving human participants and/or tissue)**<br>• Give the name of the institutional review board or ethics committee that approved the study<br>• Include the approval number and/or a statement indicating approval of this research<br>• Indicate the form of consent obtained (written/oral) or the reason that consent was not obtained (e.g. the data were analyzed anonymously)<br><br>**Animal Research (involving vertebrate animals, embryos or tissues)**<br>• Provide the name of the Institutional Animal Care and Use Committee (IACUC) or other relevant ethics board that reviewed the study protocol, and indicate whether they approved this research or granted a formal waiver of ethical approval<br>• Include an approval number if one was obtained<br>• If the study involved *non-human primates*, add *additional details* about animal welfare and steps taken to ameliorate suffering<br>• If anesthesia, euthanasia, or any kind of animal sacrifice is part of the study, include briefly which substances and/or methods were applied<br><br>**Field Research**<br><br>Include the following details if this study involves the collection of plant, animal, or other materials from a natural setting:<br>• Field permit number<br>• Name of the institution or relevant body that granted permission	
**Data Availability**<br><br>Authors are required to make all data underlying the findings described fully available, without restriction, and from the time of publication. PLOS allows rare exceptions to address legal and ethical concerns. See the PLOS Data Policy and FAQ for detailed information.	Yes - all data are fully available without restriction

A Data Availability Statement describing where the data can be found is required at submission. Your answers to this question constitute the Data Availability Statement and **will be published in the article**, if accepted.<br><br>**Important:** Stating 'data available on request from the author' is not sufficient. If your data are only available upon request, select 'No' for the first question and explain your exceptional situation in the text box.<br><br>Do the authors confirm that all data underlying the findings described in their manuscript are fully available without restriction?	
**Describe where the data may be found in full sentences. If you are copying our sample text, replace any instances of XXX with the appropriate details.**<br><br>• If the data are **held or will be held in a public repository**, include URLs, accession numbers or DOIs. If this information will only be available after acceptance, indicate this by ticking the box below. For example: *All XXX files are available from the XXX database (accession number(s) XXX, XXX.).*<br>• If the data are all contained **within the manuscript and/or Supporting Information files**, enter the following: *All relevant data are within the manuscript and its Supporting Information files.*<br>• If neither of these applies but you are able to provide **details of access elsewhere**, with or without limitations, please do so. For example:<br><br>*Data cannot be shared publicly because of [XXX]. Data are available from the XXX Institutional Data Access / Ethics Committee (contact via XXX) for researchers who meet the criteria for access to confidential data.*<br><br>*The data underlying the results presented in the study are available from (include the name of the third party*	The data are contained in the manuscript.

and contact information or URL).<br>• This text is appropriate if the data are owned by a third party and authors do not have permission to share the data.<br><br>* typeset	
Additional data availability information:	Tick here if the URLs/accession numbers/DOIs will be available only after acceptance of the manuscript for publication so that we can ensure their inclusion before publication.

Powered by Editorial Manager® and ProduXion Manager® from Aries Systems Corporation

Manuscript Click here to access/download;Manuscript;Draft_1.02.2024.docx

Impact of Climate Change on Insectivorous Bats and Implications for Small-Scale Farming in southern Mexico

Mercedes Ramírez-Barrón[1,2], Carolina Ureta[2,3†], Melanie Kolb[4], Giovanna Gasparello[5], Felipe Ruán-Soto[6], Adán L. Martínez-Cruz[7], Víctor Sánchez-Cordero[1†].

[1] Departamento de Zoología, Instituto de Biología, Universidad Nacional Autónoma de México, Circuito Exterior s/n, Ciudad de México, 04510, México.

[2] Instituto de Ciencias de la Atmósfera y Cambio Climático, Universidad Nacional Autónoma de México, Investigación Científica s/n, C.U., Ciudad de México, 04510, México.

[3] Investigadora por México-Conahcyt and member of Laboratorio Nacional de la Biología del Cambio Climático.

[4] Instituto de Geografía, Universidad Nacional Autónoma de México, Investigación Científica s/n, C.U., Ciudad de México, 04510, México.

[5] Dirección de Etnología y Antropología Social. Instituto Nacional de Antropología e Historia. Av. San Jerónimo 880, San Jerónimo Lídice, Magdalena Contreras, Ciudad de México, 10200, México.

[6] Instituto de Ciencias Biológicas, Universidad de Ciencias y Artes de Chiapas. Libramiento Norte Poniente 1150, Colonia Lajas Maciel, 29039, Tuxtla Gutiérrez, México.

[7] Department of Forest Economics, and Centre for Environmental and Resource Economics (CERE), Swedish University of Agricultural Sciences (SLU), Umeå, Sweden

[†]Corresponding author: Carolina Ureta (carolinaus@atmosfera.unam.mx); Víctor Sánchez-Cordero (victor@ib.unam.mx)

Abstract

Insectivorous bats preying on insect pests in croplands provide an important ecological service for agriculture and food security. This role is particularly relevant for small-scale farmers in Chiapas, southern Mexico, that rely on maize for subsistence. The impact of climate change is resulting in a decrease in insectivorous bat populations and an increase in insect pests. This study develops a four-step protocol to assess potential impact of climate change on insectivorous bats controlling insect pest of crops, and implications on small-scale farmers' livelihoods. We (1) used available official information to quantify costs of chemical pest control on the entire area under maize cultivation, (2) interviewed small-scale farmers to determine their perception on insectivorous bats as beneficial for preying on insect pests, (3) estimated the economic value of ecosystem services provided by insectivorous bats, and (4) projected impacts of climate change on future distributions of insectivorous bats in Chiapas. The estimated value of insect pest control by insectivorous bats that official statistics yield in Chiapas was $7.9 USD/ha for 2023. Conversely, the estimated costs resulting from interviews with small-scale farmers was $40.1 USD/ha for 2023. Most interviewed small-scale famers perceive a decline in bat population but are unaware that insectivorous bats consume large proportions of insect pests, and even fear the presence of bats in their crops. Projections of shifts in bat population due to climate change suggest potential declines of insectivorous bats in areas where small-scale farmers cultivate maize for subsistence. This scenario implied higher costs associated with chemical pest control, which in some instances may provoke that farmers abandon their crops. We highlight the complex interplay between ecosystem service (insectivorous bats controlling

insect pests), social perception of role of bats in their crops, socioeconomic factors, and small-scale agricultural sustainability.

Keywords: Bat-mediated pest control, maize, climate change, economic value[illegible]iapas.

Introduction

Species of bats are known to play an important role in providing ecosystem services such as pollination and fruit and seed dispersal of plants (including cultivated species), or consuming large quantities of insect pests of crops [1-6]. Insectivorous bats preying on insect pests actively contribute to maintain agricultural production and food security [5-8], limiting pest outbreaks, forcing insect migration, and decreasing environmental and economic costs of using large quantities of chemicals to control insect pests [9-11].

The recognition of the role of insectivorous bats in providing ecosystem services to maintain agricultural production is widely acknowledge by preying large amounts of insect pests [12]. Thus, it is important to highlight the vulnerability of species of bats to various anthropogenic factors, as loss of natural habitat and climate change [13, 14]. An effective strategy to highlight the importance of conserving species of bats providing ecosystem services is through an economic evaluation. This approach helps estimating the impact on the economic livelihood of beneficiaries [15, 16] and is particularly useful for small-scale (less than 5 hectares) agricultural farmers cultivating crops for subsistence [1-4]. Further, the State of Chiapas located in southern Mexico holds 77% of farmers in a small-scale cultivation of crops for subsistence (INEGI 2022). Chiapas holds a remarkable importance in Mexican biological and sociocultural diversity, including maize diversity and production [17]. Further, of the 138 species of bats nationwide, 70% are insectivorous bats of which 67 species occur in Chiapas [18].

Quantifying and assigning economic value to insectivorous bats preying on insect pests as an ecosystem service has been conducted through experimental exclusion, synchronization between pest and predator cycles, dietary preferences, and the avoided

costs model [10, 12, 19-21]. The avoided cost model is based on methods where the expense of chemical pest control serves as a baseline reference [22-24]. While these contributions are essential in the economic evaluation, recognizing the importance of conserving insectivorous bat populations for their ecological contributions must also be included. Thus, evaluating the impact of anthropogenic factors as loss of habitat or climate change provide relevant information on the potential consequences of disruption of ecosystem services provided by bats [13]. Understanding the complex interaction between ecosystem services and their associated threats can provide insights of incentives for the conservation of bat species and the continuity of benefits for small-scale farmers cultivating crops for subsistence [25]. Here, we combined quantitative and qualitative methods to conduct an economic evaluation in small-scale field crops in Chiapas considering the potential impacts of climate change on insectivorous bats and the perceptions of small-scale farmers regarding this ecosystem service (Fig 1). We aimed to contribute to a comprehensive understanding including an economic evaluation, as well as an ecological and social facet critical for effective conservation efforts.

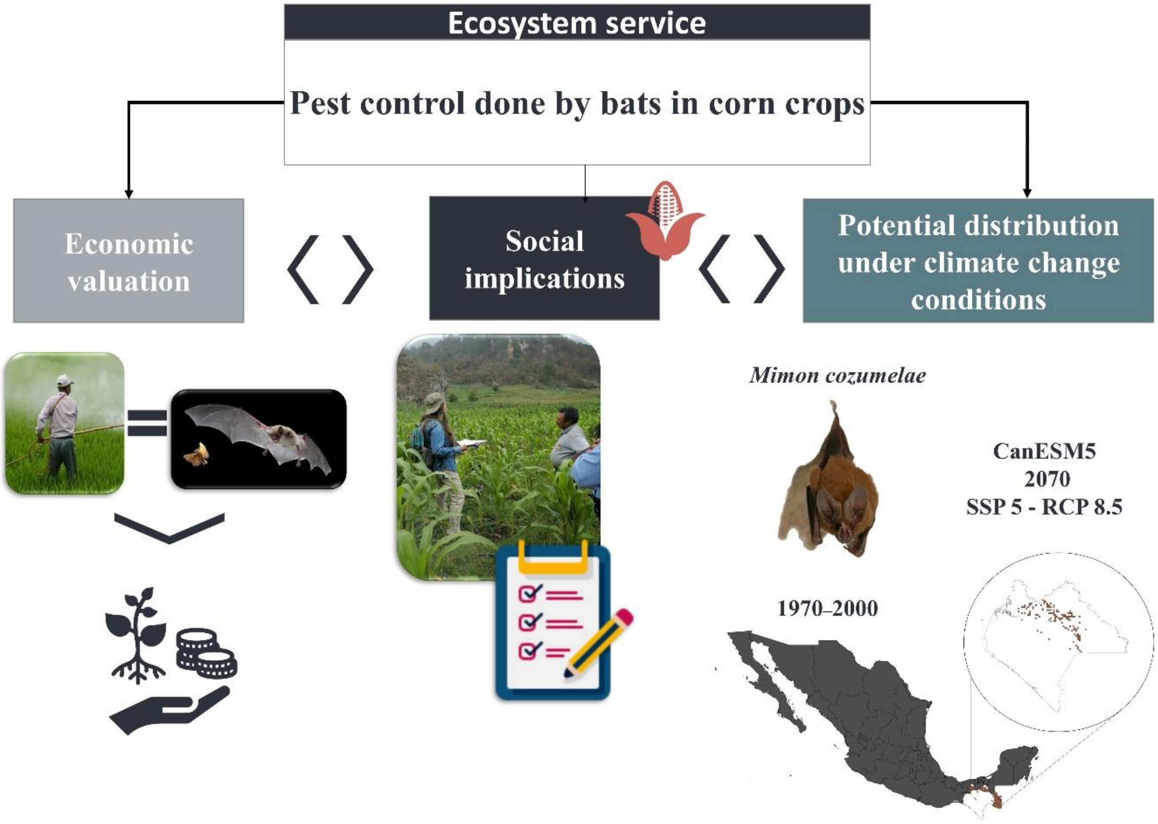

Figure 1. Flowchart illustrating the methodological approach used in this study, including economic, environmental, and social factors. We illustrate the case of the insect-eating bat species *Mimon cozumelae*, as an example. First, we conducted an economic valuation of pest control using the avoided cost approach, utilizing data on the areas of cultivated maize and expenditures related to chemical controls. Then, we integrated the insect-eating bat species modeled in Ureta et al. 2022 using three time series (2030, 2050, and 2070) and the general circulation models CanESM5 and BCC-CSM2.MR under the scenarios SSP5-RCP 8.5 W/m^2 and SSP2-RCP 4.5 W/m^2. This allowed us to estimate the average suitable area gained or lost for *M. cozumelae* in Chiapas. Lastly, we depicted the value of bat-mediated pest control for the surveyed families, showcasing their perception of bats and their significance of cultivating maize.

Materials and Methods

Our methodological approach incorporated a four-stage protocol (1) using official information on maize cultivation and use of chemical pesticide, (2) conducting on-site interviews with rainfed and irrigated small-scale farmers for Chiapas in Southern Mexico, (3) integrating information from official sources and interviews to estimate the value of the ecosystem service provided by native maize, employing the avoided cost methodology, and also gaining insights into community perceptions, and (4) using of a correlative approach to project the distribution shifts of bats under climate change scenarios in Chiapas (Fig 1).

Bat potential distribution models

A total of 67 species of distribution models of insectivorous bats were used from published database [14] with distribution projections of bats occurring in Chiapas were used for our study. The distribution models were built based on machine-learning algorithms that associate environmental factors (climate-related variables) with species georeferenced records and projected as species potential distributions [26]. Only species of bats with a minimum of 25 records (10 km-spaced) were modeled to ensure data for model calibration (70%) and validation (30%), following published protocols [27]. A set of algorithms, including MaxEnt, GBM, GLM, GAM, CTA, and RF were used, and validation metrics included Kappa, TSS, and ROC. A total of nineteen bioclimatic variables at a resolution of 10 km^2 were included [28], capturing annual trends and temporal fluctuations of climatic values with biological relevance were included. For each species, a preliminary variable selection was conducted using a Pearson test ($r < 0.8$). We used species distribution models projected to the following scenarios: (1970–2000), 2030 (2021–2040), 2050 (2041–2060),

and 2070 (2081–2100). We integrated two distinct general circulation models: CanESM5 (CAN), recognized for its accurate simulation of Mexican climate, and BCC-CSM2.MR (BCC) selected due to its climatic disparity from CAN (being colder and drier). This juxtaposition of contrasting models was aimed to consider the climatic variation Furthermore, each model corresponds to different socioeconomic pathways, with implementations under both SSP2-RCP 4.5 (SSP 245) representing moderate development; and SSP5-RCP 8.5(SSP585) reflecting a fossil fuel-dependent development trajectory [29, 30] [please go to 14 for further methodological details].

Socioeconomic impact

This study implemented the avoided costs method (ACM) for estimating the economic value of insectivorous bat controlling insect pest on maize crops. This approach is used in cases for which environmental services lack a market, but their value can be inferred through existing markets of good or services that effectively work as substitutes of or complements to ecosystem services [31, 32]. In implementing the ACM, we assumed that a[illegible]pecies of insectivorous bats contribute equally to pest control and serving as important consumers of insects damaging maize crops. Our approach was initially based on the cost of chemical pest control at the State level [33]. The cost of a single unit of chemical pest control to be $15.15 USD – numbers in USD are calculated based on the average exchange rate for the first eleven months of 2023, provided by the Mexican Central Bank (17.81 MXN/USD) –. This estimate resulted from the price of chemical pest control per ha at the State level from FIRA [34], and then multiplied by the number of units corresponding to the cultivated area of corn in Chiapas [one unit is

678,590.13 ha from 35], which according to SIAP [36] only uses chemical controls in 52.39%. That is,

Insect pest control by insectivorous bats = CHPCH × SSM × CHPCUF,

where:

CHPCH is the cost of chemical pest control per ha of Chiapas ($15.2USD); SSM is the corn cultivated area (678,590.13 ha), and CHPCUF is the chemical pest control use factor (0.5239%).

Interview protocol

The interviews helped to complement official data with local knowledge about costs and gaining understanding about farmers perceptions to insectivorous bats. For our interview selection process, we used official data to identify geographical cells of 10 km^2 with highest area of maize crop and bat species richness. The localities visited were: Chatetic (Chilón Municipality), Navenchauc (Zinacantán Municipality), San Isidro Chijilté (Teopisca Municipality) and the Muncipal headlands of Amatenango del Valle, Teopisca, Venustiano Carranza, Palenque, San Juan Chamula and Socoltenango (Fig 2). To take the interviews, there was always verbal consent and were carried out voluntarily. We have a local university student that witnessed all interviews that were carried out and helped with language translation. The villages sampled have an indigenous Tsotsil, Tseltal and Ch'ol population as well as mestizo people. We employed the snowball sampling method to identify suitable interviewees, which consists in identifying individuals who meet specific criteria, such as ethnicity, occupation

(traditional peasants), and then relying on their input to identify additional participants [37]. The snowball sampling is very useful in qualitative studies to identify key informants who have characteristics and knowledge relevant to the research, especially in small, bounded, or difficult to find populations [38]. A structured interview [sensu 38] was conducted with the informants allowing a deeper understanding of the perception of small-scale farmers regarding the role of bats, their cultural significance, and the strategies they employ to adapt to climate change scenarios. The insights compiled from these interviews provided a detailed local information about the economic value of the ecosystem service in these communities. The interviews protocol is described in Supplementary Material I.

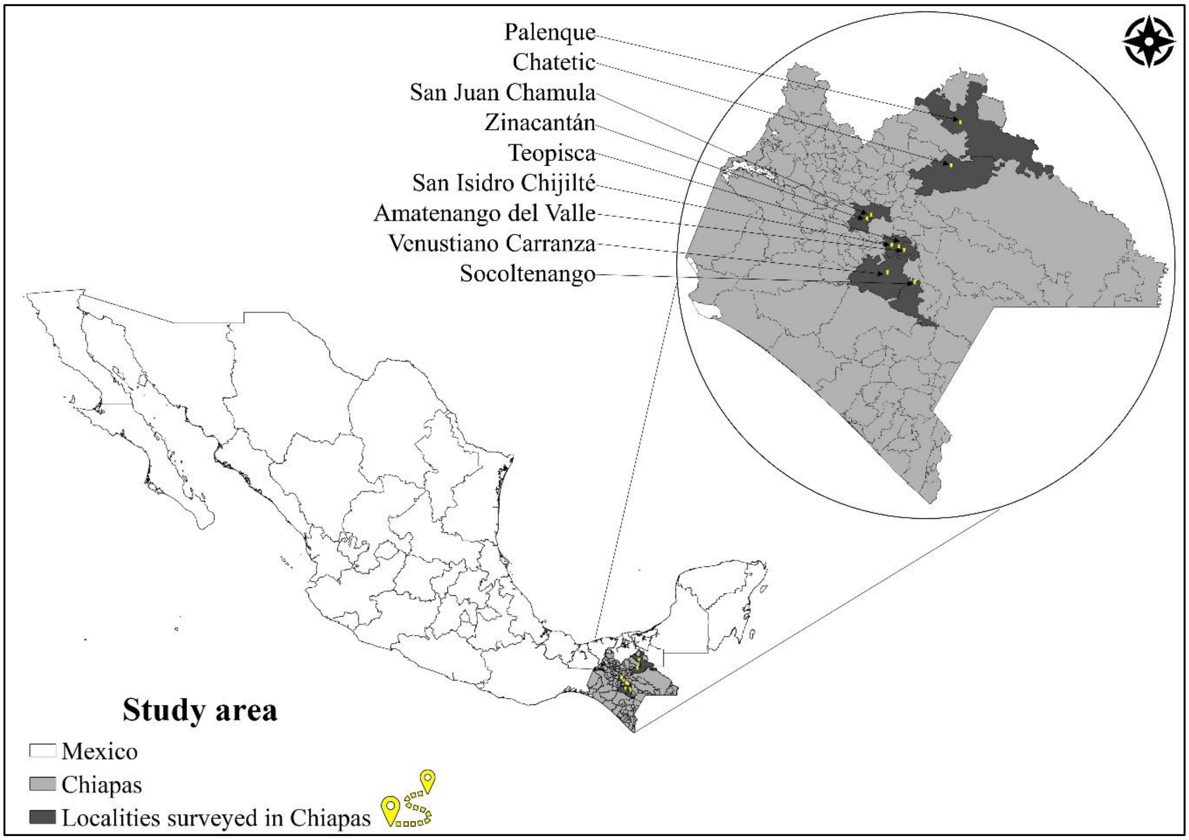

Figure 2. Study area. Surveyed localities (Amatenango del Valle, San Isidro Chijilté,

Teopisca, Chatetic, Zinacantán, Venustiano Carranza, Palenque, San Juan Chamula and Socoltenango) in Chiapas (highlighted in yellow on the map).

Results

Bats distributions models

We built distribution models of 67 species of insectivorous bats occurring in Chiapas [18] under baseline and future climate change scenarios occurring in Chiapas [see 14]. Under the non-dispersal assumption (without considering new suitable areas that might appear in different geographic regions), the most pronounced suitability declines in Chiapas are represented by SSP585 for the year 2070 (2081–2100) (Fig 3, Table 1). In this scenario, species such as *Balantiopteryx io, Eumops auripendulus, Glyphonycteris sylvestris, Lonchorhina aurita*, *Lophostoma brasiliense*, *Perimyotis subflavus*, *Peropteryx macrotis*, *Phylloderma stenops*, *Pteronotus gymnonotus*, *Rhogeessa genowaysi*, and *Thyroptera tricolor* experienced suitability losses ranging from 73% to 100%[illegible]he species at the highest climatic risk in most evaluated scenarios were *Balantiopteryx io* (73% to 100%), *Mimon crenulatum* (> 90% suitability loss in three out of four climate change scenarios in 2030), and *Rhogeessa genowaysi* (70-88% suitability loss under all SSP585 2050 and 2070 scenarios) (Fig 3, Table 1). In general, the highest suitability losses for most species were observed in the time horizon 2070 and scenario SSP585 characterized by pessimistic fossil fuel emission projections; and general circulation mode BCC that represents colder but drier conditions compared to CAN.

Under the full-limited dispersal assumption, gains in climatic suitability outweighed losses for all insect-eating species of bats in the 2030, 2050, and 2070 evaluated scenarios. The only exception was the SSP245 BCC in 2030, where losses were slightly higher (5%) than gains in climatic suitability. Substantial gains are expected under the general circulation model CAN, which forecasts warmer and more humid conditions than BCC. Our results suggest that, assuming limited-full dispersal ability across Chiapas, most species are likely to find new areas with suitable climate conditions (Table 2). Under the assumption of species limited-full dispersal, the descending trend in suitability over time persists, particularly pronounced under SSP245 CAN (Table 1). The suitability of insectivorous bats in highly diverse regions near the Pacific Coastal Plain was examined under both baseline and climate change scenarios. Despite the presence of the species *Eumops hansae*, *Molossus aztecus*, and *Rhogeessa aeneus* in Chiapas, these three species did not exhibit climatic suitability under the baseline scenario for the period 1970–2000 and were excluded from the analyses.

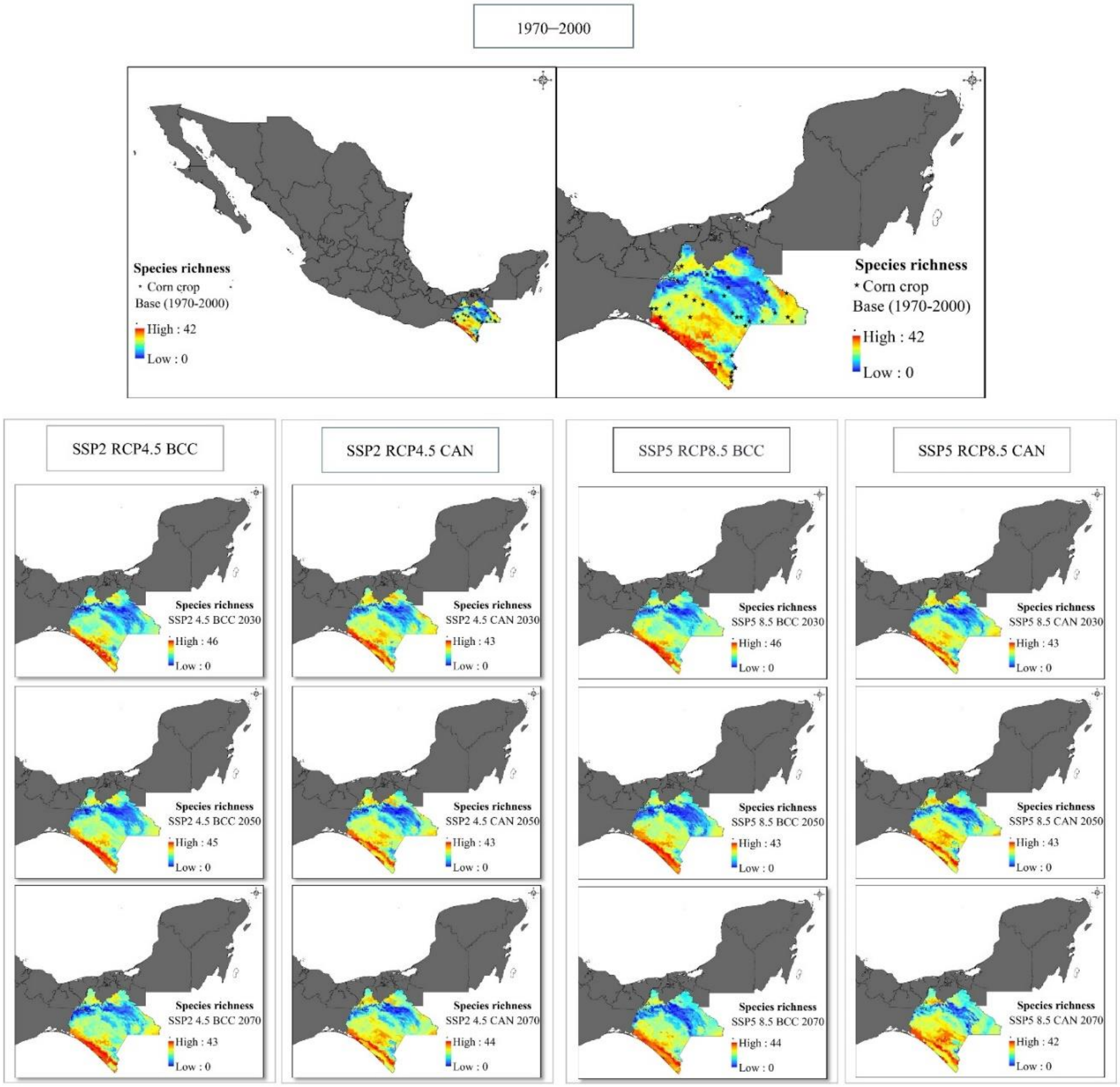

Figure 3. Geographical representation of bat species richness areas within Chiapas in the baseline scenario (1970-2000) and under climate change scenarios (2030, 2050, and 2070) using general circulation models CanESM5 and BCC-CSM2.MR under the scenarios SSP5-RCP 8.5 W/m² and SSP2-RCP 4.5 W/m². Under the baseline scenario it is also possible to identify the localities with higher maize richness.

Table 1. Species potential distributions estimated for all climate change scenarios and general circulation models in the 2030, 2050, and 2070 projections for the insectivorous bats present in Chiapas. SPD = potential distributions of species; Models SSP2-RCP4.5-BCC, SSP2-RCP4.5-CAN, SSP5-RCP8.5-BCC and SSP5-RCP8.5-CAN included in the analysis. The SPDs were estimated under a full-limited and no-dispersal assumptions, respectively.

**SPS limited full dispersal**						
		**245_BCC**	**245_CAN**	**585_BCC**	**585_CAN**	**Range**
2030	Mean	0.170	0.147	0.180	0.170	0.147–0.18
	Median	0.136	0.080	0.130	0.128	0.08–0.136
2050	Mean	0.178	0.191	0.293	0.251	0.178–0.293
	Median	0.111	0.085	0.261	0.123	0.085–0.261
2070	Mean	0.223	0.222	0.334	0.224	0.222–0.334
	Median	0.174	0.094	0.279	0.094	0.094–0.279
**SPD non-dispersal**						
2030	Mean	-0.003	0.087	0.036	0.045	-0.003–0.087
	Median	0.000	0.025	0.027	0.035	0–0.035
2050	Mean	0.257	0.143	0.020	0.139	0.02–0.257
	Median	0.007	0.035	-0.058	0.000	-0.058–0.035
2070	Mean	0.076	0.190	0.125	0.196	0.076–0.196
	Median	0.004	0.031	-0.012	0.031	-0.012–0.031

Table 2. Percentage of interviewed indigenous small-scale farmers ISF), categorized by language, who are aware (% Yes) and unaware (% No) of the ecosystem service provided by insect-eating bats in pest control of maize crops in the sampled localities in Chiapas.

ISF	Yes (%)	No (%)	Do not know (%)
Ch’ol	–	–	2
Mestizo	11	13	–
Tseltal	6	40	13
Tsotsil	2	11	2
**Total**	**19**	**64**	**17**

Interviews of farmers

A total of 77% of farmers in Chiapas are small-scale farmers that cultivate for subsistence purposes [39]. In March 2020, we conducted 53 interviews with small-scale farmers from various indigenous groups in Chiapas, including 58% Tseltales, 25% Mestizos, 15% Tsotsiles, and 2% Choles. Of those interviewed, 79% were male and 21% female (Table 2). Half of the surveyed farmers reported using a similar portion of their maize harvest for personal consumption and for selling. The income generated from selling maize helps farmers purchase essential cooking supplies (like oil and sugar) and personal items (such as soap). Approximately 38% of respondents stated that their maize harvests are solely for self-consumption, and 13% of farmers cultivate maize for selling. Maize cultivation primarily aims to meet the basic needs of families (ranging from 2 to 11 individuals).

Further, 62% of farmers mentioned that they have not observed bats in their crops, 14% have reported observing them, and 6% were uncertain. Those who mentioned not observing bats in their crops or being unsure explained that this might be because their work hours are from 7:00 to 18:00 and bats are nocturnal. On the other hand, those who have observed bats in their maize crops do not perceive them as beneficial for their maize fields. One third of farmers believe that bats are consuming their corn, while 19% mentioned that bats contribute positively

to their crops by aiding in seed dispersal, pollination, or preying on insect pests (7%). Additionally, 17% expressed uncertainty regarding the role of bats in their crops. Furthermore, Tseltal farmers (40%) are the least familiar with the role of bats as consumers of insect pests, whereas Mestizo farmers (11%) were the most aware of the benefits of bats preying on insect pests (Table 2). Further, when we asked farmers if they have observed more or fewer bats in the last year compared to previous years, 60% indicated that they have observed fewer bats in the area. In contrast, 21% perceived an increase in the bat population, while 15% mentioned that they have not seen any bats, and 2% believed that the number of bats remains the same. The farmers attribute the fluctuation in the bat population primarily to deforestation, climate change in the region, and human-induced mortality.

When asked about purchasing more pesticides due to an increase in insect pests of corn, 17% of farmers expressed the ability to afford such products for pest mitigation, 7% were uncertain about their ability to make the purchase, and 30% mentioned they would not be able to afford the products. Those who indicated they could not purchase more pest mitigation products mentioned that they would have to seek for loans, find ways to save money, or even consider not planting corn at all (Fig 4). Lastly, we inquired about the farmers' responses if they had to stop cultivating maize. The majority indicated that they would try to cultivate a different crop (36%), while 32% of farmers mentioned they would not have food. Some farmers considered the option of changing their occupation (15%) or purchasing maize (7%), but others were considering abandoning their land (6%) (Fig 5).

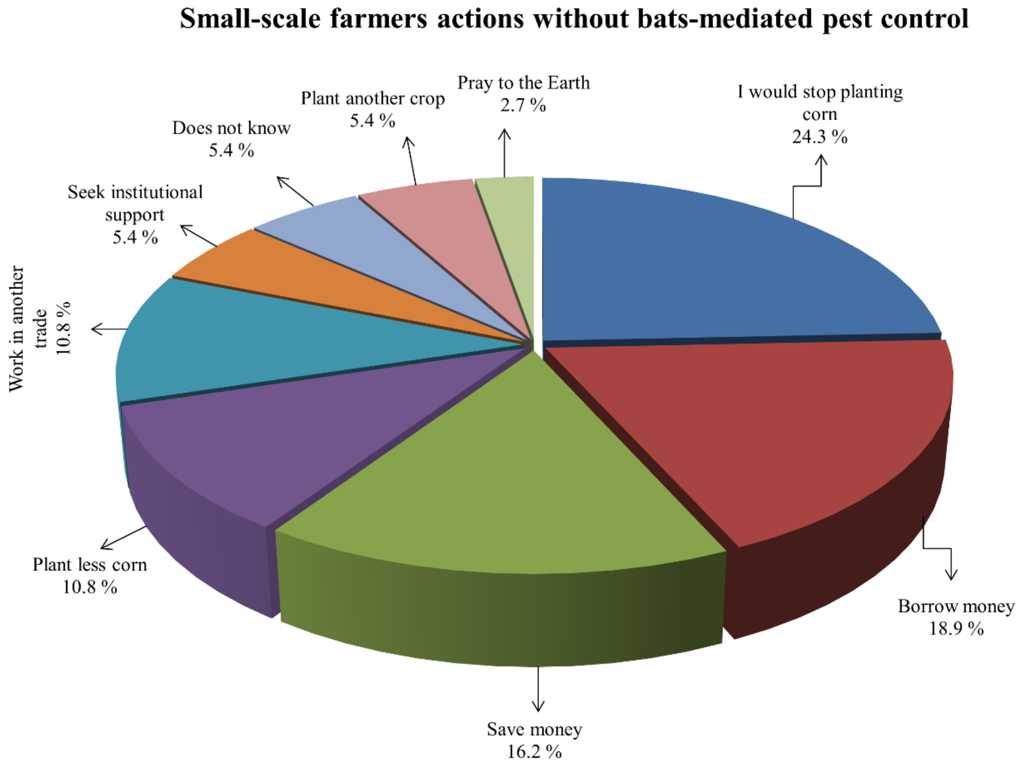

Figure 4. Responses provided by the small-scale farmers regarding the scenario of actions they would take if they did not have the funds to acquire products or a larger quantity of those products due to increased pests in maize cultivation.

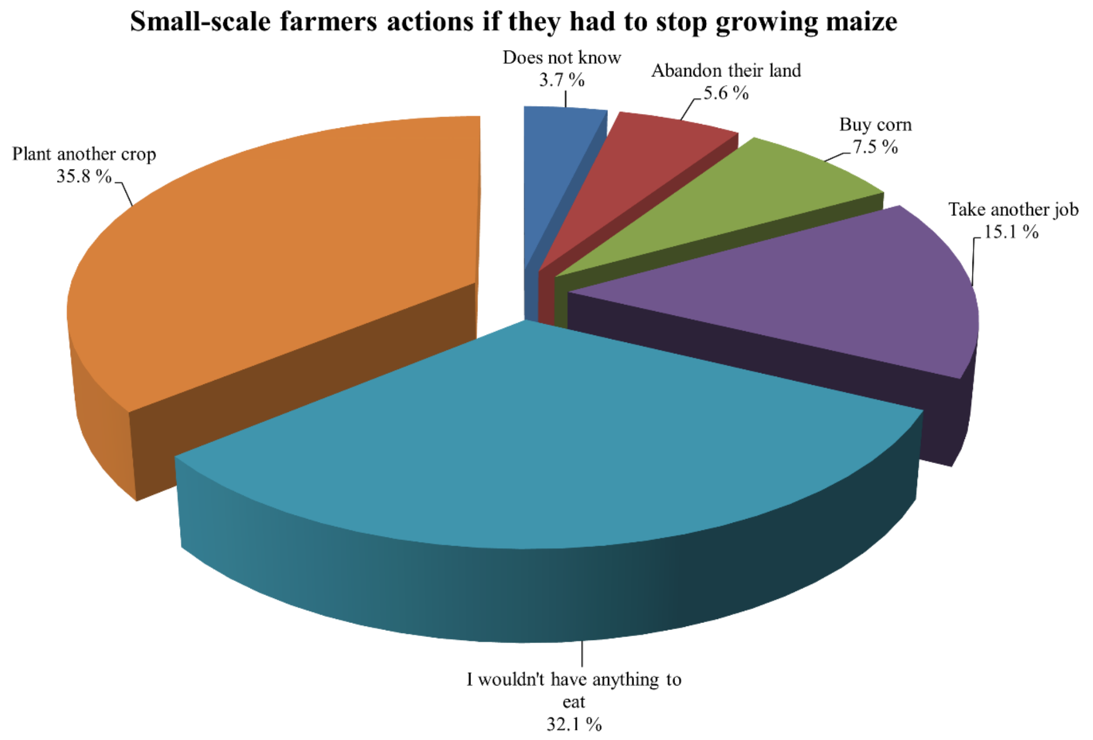

Figure 5. Small farmers surveyed. Responses provided by the small-scale farmers regarding the scenario in which they would have to stop cultivating maize.

Socioeconomic evaluation

Based on the interviews, we estimated that small-scale farmers cultivated approximately 104.1 ha of maize annually and spent $4,813 USD per year on acquiring chemical compounds for pest control in their maize (such as urea, carioca, hydrochloric acid, and foley). This corresponds to a weighted expenditure of approximately $40.1 USD/ha of cultivated land for the agricultural year. Moreover, our findings revealed that even when 81% of the small-scale farmers employ chemical pest controls in maize cultivation, 15% do not use any form of pest

control, and the remaining 4% use biological control methods (such as fish waste or chili-infused salt). It is important to mentioned that insectivorous bats also consume other invertebrates strongly affecting maize crops as the white grub (gallina ciega), armyworm (gusano soldado), corn earworm (gusano cogollero), and ants, among others [3].

We estimated approximately 52.39% of the cultivated area using chemical pest control in the region, which is equivalent to 355,513.4 ha [35]. This percentage corresponds to pesticide use according to the 2019 National Agricultural Survey, resulting in an estimated service value of $5’387,408.1 USD per agricultural year for rainfed maize protection in Chiapas. In terms of surface area, the estimate amount was $7.9 USD per ha.

Discussion

Future rising temperatures due to climate change may lead to an increase in the number of insects infesting maize crops [40]. If so, insect infestations increase, combined with a lower presence of insectivorous bats in crops, will potentially lead to higher economic impacts on small-scale farmers. Furthermore, insectivorous bats show population declines in degraded and fragmented habitats [41-43]. Most farmers in this study have noticed fewer bats in their cultivated lands (Table 2), which is probably due to a rampant deforestation in Chiapas [44]. This perception is consistent with land use transformations, a reduction in climate suitability, and other economic activities in the region –all three documented factors of reduction in bats populations [45]. This scenario highlights the complex interplay of climate, ecosystem services, biodiversity conservation, and socioeconomic factors on crop production, and urges for an integrated pest control management in accordance with the conservation of insectivorous bats in the region.

Our study reveals that projected climatic suitability of insectivorous bats will strongly depend on their ability to disperse to new areas (Fig 3, Table 1). Thus, insectivorous bats might encounter challenges in reaching these areas depending on the landscape connectivity and suitability of habitats. If insectivorous bats manage to disperse to new climatically suitable areas shifting their distribution, this might result in a population decrease in current areas of cultivated maize.

We identified that cultivation of maize holds high significance in the nutrition, culture, and economy of families in the surveyed areas. Local small-scale farmers emphasized their strong reliance on cultivation of maize to feed their families, in part because their culture and traditions are deeply rooted in consuming this crop as an essential component of their diet [46, 47]. Agriculture in this region falls primarily for family consumption, and any commercial activities are conducted for subsistence and accessing other basic goods [48]. Our interviews helped us highlight that small stakeholders engaged in maize trade also hire individuals who benefit from wages, also contributing to cover the basic needs of other community members. We also detected that while cultivating has been traditionally conducted by men, there is a gradual shift of more participation of women in this activity. The interviews show that women have increased their participation in the last decades due to decreasing yields [49] of maize in the area, and emigration of men seeking for better economic opportunities [50, 51]. This has prompted the involvement of most family members to cultivation activities to enhance crop productivity.

The agricultural landscape of southern Mexico has experienced significant increases in land-use cover changes as well as deterioration in the economy of farmers by the transition toward neoliberal agriculture models. This transition entails the displacement of traditional production methods and the deterioration of ecological conditions in the studied localities [52,

53]. In this context, we observed a decline or loss of traditional knowledge about maize cultivation, with only elderly individuals recognizing the significance of insectivorous bats in eradicating insect pests from their maize crops. In contrast, younger generations are unaware of the role insectivorous bats play in their crops. Unfortunately, this reflects the cultural erosion that has emerged from changing traditional methods to a modernization of agriculture [54]. The modernization of agriculture has resulted in the intensive use of chemical pest controls to maintain maize crop yields [55, 56], putting aside traditional practices that included natural selective weeding [57].

Further, the excessive use of chemical pest controls without a sound integrated pest management protocol has produced a deterioration of both social and ecosystems health [58-60]. Our results suggest that the overuse of chemical controls by small-scale farmers cultivating maize is linked to inadequate knowledge and improper handling of these products. The lack of appropriate information leads to increased health risks for farmers and their families, and other non-target species including insectivorous bats [60, 61]. Although many bat species can thrive in landscapes transformed by agriculture [62], exposure to chemical pests poses a growing threat increasing the vulnerability of these species to environmental changes [63, 64]. This situation poses a risk to the ecosystem service that insectivorous bats provide, as chemical controls used in agriculture have been linked to declines in bat populations due to poisoning [65]. Lastly, we also detected a language barrier between farmers belonging to different ethnic groups, hindering access to information about the importance of insectivorous bats. Our study suggest that Mestizo farmers readily recognize the benefits provided by insectivorous bats, possibly due to their access to information in their language, compared to indigenous language-speaking farmers.

We found that insectivorous bats mediating as insect pest of crops play a crucial role in supporting the production and sustenance of rural families, based on our socioeconomic assessment. However, most farmers appear to have a lack of awareness about the value of ecosystem services insectivorous bats provide to their crops. This lack of understanding of farmer of the role of insectivorous bats on preying on insect pests on maize crops limits strategies on food security, conservation of maize diversity, and bat species richness. To overcome these adversities, it is imperative to reconcile traditional knowledge of farmers on cultivation of maize, and convincing of the important role of insectivorous bats preying on insect pests of crops, as an essential ecosystem service.

The cost of chemicals to control insect pests purchased by farmers allowed to quantify the value of the ecosystem service provided by bat-mediated insect pest control as a partial substitute for such control methods. Calculations using available official data yield $7.9 USD/ha as the value of bat-mediated insect pest control in Chiapas. However, calculations based on data reported by farmers participating in our study yield an estimate of $40.1 USD/ha. This value is about five times higher in comparison to other regions. For example, Gándara et al. [66] estimated the cost of pest control in maize ranging between 8.3 and $18.6 USD/ha in Nuevo León, in northern Mexico, based on pesticide applications. Maine & Boyles [23] who considered maize yield and price to assess by bat-mediated pest control at $7.9 USD/ha in the United States. These studies align to what can be inferred with official information in Chiapas – however, these studies lack firsthand information that our study has obtained directly from small-scale farmer families cultivating maize in Chiapas—. On the other hand, Aguiar et al. [24] found that the ecosystem service value of insect pest suppression by insectivorous bats saves farmers $94 USD/ha, reaching an annual savings of $390.6 million per harvest in Brazil.

In sum, we observed that a decline in the presence of bat-mediated insects pests on maize crops will have negative economic and food sufficiency implications for subsistence agriculture of small-scale farmers in Chiapas. At the same time, we have documented that although the majority of interviewed famers perceive a decline in bat population, they do not perceive bats as providers of pest control services –with some farmers reporting fear to presence of bats in their crops—. This perception represents a challenge to promote protection of bats. Alternatives to overcome such challenge include the design of programs that may offer farmers compensation –either monetary or non-monetary or both— in exchange of, for instance, a reduction in use of chemical fertilizers so that bat population is preserved. In a similar vein, it has been documented that agave producers in Jalisco, Mexico, report interest in a (hypothetical) program that may compensate them for measures aiming to improve both genetic conservation of blue agave and protection of magueyero bats [67]. As the design of such type or programs may take time and political will, meanwhile we recommend local governments and biologists to provide practical information to farmers on the importance of the ecosystem services provided by insectivorous bats preying on insect pests on crops. Moreover, an integrated pest management protocol should be established for an adequate use of chemicals to control pests on crops [68-70]. We are aware that our study presents a preliminary approximation of the value of insectivorous bats preying on insect pests of maize crops as an ecosystem service in Chiapas. Our values are a conservative estimation as not all insectivorous bat species contribute equally to agricultural insect pest control. Further research effort is needed to understand the relationship between each insectivorous bat species and maize pests. We also emphasize that sustaining maize cultivation in the study areas facilitates food security, particularly considering limited access to sufficient and nutritious food among the surveyed farmers. Many farmers are indigenous and non-Spanish speakers, facing difficulties in

obtaining institutional support. The language barrier further reduces their access to information about the importance of insectivorous bats; mestizo communities which are knowledgeable on Spanish recognize the role of insectivorous bats more readily (Table 2). Detrimental factors as climate change and land-use cover changes could profoundly impact the conservation of insectivorous bats [14[illegible] The loss of maize crops translates to the loss of other subsistence agriculture systems, limiting the likelihood of achieving food sufficiency, food sovereignty, and its sociocultural nature-based self-identity in the region.

Acknowledgements

We extend our gratitude to the Postgraduate Program in Sustainability Sciences of the National Autonomous University of Mexico (UNAM) and CONACYT for the postgraduate studies of the first author, Mercedes Ramírez-Barrón. Likewise, we appreciate the guidance and technical support provided for the economic valuation by Juan Gerardo Juárez Hermosillo, Graduate in Financial Administration. We also appreciate the field support provided by Biologist Isabel Vanessa Flores Sánchez for helping with the interviews.

References

1. Kunz TH, Braun de Torrez E, Bauer D, Lobova T, Fleming TH. Ecosystem services provided by bats. Annals of the New York academy of sciences. 2011;1223(1):1-38.
2. Williams-Guillén K, Olimpi E, Maas B, Taylor PJ, Arlettaz R. Bats in the anthropogenic matrix: challenges and opportunities for the conservation of Chiroptera and their ecosystem services in agricultural landscapes. Bats in the Anthropocene: Conservation of bats in a changing world. 2016:151-86.
3. Whitby MD, Kieran TJ, Glenn TC, Allen C. Agricultural pests consumed by common bat species in the United States corn belt: the importance of DNA primer choice. Agriculture, Ecosystems & Environment. 2020;303:107105.
4. Allen L, Richardson C, McCracken G, Kunz T. Birth size and postnatal growth in cave- and bridge- roosting Brazilian free- tailed bats. Journal of Zoology. 2010;280(1):8-16.
5. Ghanem SJ, Voigt CC. Increasing awareness of ecosystem services provided by bats. Advances in the Study of Behavior. 44: Elsevier; 2012. p. 279-302.
6. Baroja U, Garin I, Vallejo N, Aihartza J, Rebelo H, Goiti U. Bats actively track and prey on grape pest populations. Ecological Indicators. 2021;126:107718.
7. Leelapaibul W, Bumrungsri S, Pattanawiboon A. Diet of wrinkle-lipped free-tailed bat (Tadarida plicata Buchannan, 1800) in central Thailand: insectivorous bats potentially act as biological pest control agents. Acta Chiropterologica. 2005;7(1):111-9.
8. Williams-Guillén K, Perfecto I, Vandermeer J. Bats limit insects in a neotropical agroforestry system. Science. 2008;320(5872):70-.
9. Lee Y-F, McCracken GF. Dietary variation of Brazilian free-tailed bats links to migratory populations of pest insects. Journal of Mammalogy. 2005;86(1):67-76.
10. Cleveland CJ, Betke M, Federico P, Frank JD, Hallam TG, Horn J, et al. Economic value of the pest control service provided by Brazilian free- tailed bats in south- central Texas. Frontiers in Ecology and the Environment. 2006;4(5):238-43.
11. Ramírez- Francél LA, García- Herrera LV, Losada- Prado S, Renoso- Flórez G, Sánchez- Hernández A, Estrada-Villegas S, et al. Bats and their vital ecosystem services: a global review. Integrative Zoology. 2022;17(1):2-23.
12. Rodríguez-San Pedro A, Allendes JL, Beltrán CA, Chaperon PN, Saldarriaga-Córdoba MM, Silva AX, Grez AA. Quantifying ecological and economic value of pest control services provided by bats in a vineyard landscape of central Chile. Agriculture, Ecosystems & Environment. 2020;302:107063.
13. Sherwin HA, Montgomery WI, Lundy MG. The impact and implications of climate change for bats. Mammal Review. 2013;43(3):171-82.
14. Ureta C, Ramírez- Barrón M, Sánchez- García EA, Cuervo- Robayo AP, Munguía- Carrara M, Mendoza- Ponce A, et al. Species, taxonomic, and functional group diversities of terrestrial mammals at risk under climate change and land- use/cover change scenarios in Mexico. Global Change Biology. 2022;28(23):6992-7008.
15. Boyles JG, Cryan PM, McCracken GF, Kunz TH. Economic importance of bats in agriculture. Science. 2011;332(6025):41-2.
16. López-Hoffman L, Wiederholt R, Sansone C, Bagstad KJ, Cryan P, Diffendorfer JE, et al. Market forces and technological substitutes cause fluctuations in the value of bat pest-control services for cotton. PLoS One. 2014;9(2):e87912.

17. Boege E, Chan GV. El patrimonio biocultural de los pueblos indígenas de México: hacia la conservación in situ de la biodiversidad y agrodiversidad en los territorios indígenas. Instituto Nacional de Antropología e Historia México, 2008.
18. Sánchez-Cordero V, Botello F, Martínez JJF, Rodríguez RAG, Guevara L, Granados GG, Moreno ÁR. Biodiversidad de Chordata (Mammalia) en México. Revista Mexicana de Biodiversidad. 2014;85:27.
19. Betke M, Hirsh DE, Makris NC, McCracken GF, Procopio M, Hristov NI, et al. Thermal imaging reveals significantly smaller Brazilian free-tailed bat colonies than previously estimated. Journal of Mammalogy. 2008;89(1):18-24.
20. Federico P, Hallam TG, McCracken GF, Purucker ST, Grant WE, Correa-Sandoval AN, et al. Brazilian free- tailed bats as insect pest regulators in transgenic and conventional cotton crops. Ecological Applications. 2008;18(4):826-37.
21. Wiederholt R, López-Hoffman L, Svancara C, McCracken G, Thogmartin W, Diffendorfer JE, et al. Optimizing conservation strategies for Mexican free-tailed bats: a population viability and ecosystem services approach. Biodiversity and Conservation. 2015;24:63-82.
22. Gándara G, Correa A, Hernández C. Valoración económica de los servicios ecológicos que prestan los murciélagos Tadarida brasiliensis como controladores de plagas en el norte de México. Cátedra de Integración Económica y Desarrollo Social, Tecnológico de Monterrey. 2006.
23. Maine JJ, Boyles JG. Bats initiate vital agroecological interactions in corn. Proceedings of the National Academy of sciences. 2015;112(40):12438-43.
24. Aguiar LM, Bueno-Rocha ID, Oliveira G, Pires ES, Vasconcelos S, Nunes GL, et al. Going out for dinner—The consumption of agriculture pests by bats in urban areas. PLoS One. 2021;16(10):e0258066.
25. Jones G, Jacobs DS, Kunz TH, Willig MR, Racey PA. Carpe noctem: the importance of bats as bioindicators. Endangered species research. 2009;8(1-2):93-115.
26. Anderson RP, Martínez-Meyer E, Nakamura M, Araújo MB, Peterson AT, Soberón J, Pearson RG. Ecological niches and geographic distributions (MPB-49): Princeton University Press; 2011.
27. Pearson RG, Raxworthy CJ, Nakamura M, Townsend Peterson A. Predicting species distributions from small numbers of occurrence records: a test case using cryptic geckos in Madagascar. Journal of biogeography. 2007;34(1):102-17.
28. Fick SE, Hijmans RJ. WorldClim 2: new 1- km spatial resolution climate surfaces for global land areas. International journal of climatology. 2017;37(12):4302-15.
29. O'Neill BC, Kriegler E, Riahi K, Ebi KL, Hallegatte S, Carter TR, et al. A new scenario framework for climate change research: the concept of shared socioeconomic pathways. Climatic change. 2014;122:387-400.
30. IPCC. Climate Change 2021: The Physical Science Basis. Contribution of Working Group I to the Sixth Assessment Report of the Intergovernmental Panel on Climate Change. Masson-Delmotte V, P. Zhai, A. Pirani, S.L. Connors, C. Péan, S. Berger, N. Caud, Y. Chen, L. Goldfarb, M.I. Gomis, M. Huang, K. Leitzell, E. Lonnoy, J.B.R. Matthews, T.K. Maycock, T. Waterfield, O. Yelekçi, R. Yu, and B. Zhou, editor. Cambridge, United Kingdom and New York, NY, USA: Cambridge University Press; 2021.

31. Cristeche E, Penna JA. Métodos de valoración económica de los servicios ambientales. Estudios socioeconómicos de la sustentabilidad de los sistemas de producción y recursos naturales. 2008;3:1-58.
32. Sundberg S. Replacement costs as economic values of environmental change: A review and an application to Swedish sea trout habitats: Citeseer; 2004.
33. Shabman LA, Batie SS. Economic value of natural coastal wetlands: a critique. Coastal Management. 1978;4(3):231-47.
34. FIRA. Panorama agroalimentario/Fríjol. Fideicomisos Instituidos en Relación con la Agricultura; 2016.
35. INEGI. Porcentaje de unidades de producción con agricultura a cielo abierto según tipo de tecnología por entidad federativa. In: Geografía INdEy, editor. Ciudad de México2019.
36. SIAP. Anuario estadístico de la producción agrícola. In: Pesquera SdIAy, editor. Ciudad de México2021.
37. Ballestín González B, Fàbregues Feijóo S. La práctica de la investigación cualitativa en ciencias sociales y de la educación: Editorial UOC; 2019.
38. Bernard HR. Research methods in anthropology: Qualitative and quantitative approaches: Rowman & Littlefield; 2017.
39. INEGI. Censo Agropecuario. In: Geografía INdEy, editor. Ciudad de México2022.
40. Deutsch CA, Tewksbury JJ, Tigchelaar M, Battisti DS, Merrill SC, Huey RB, Naylor RL. Increase in crop losses to insect pests in a warming climate. Science. 2018;361(6405):916-9.
41. Medellín RA, Equihua M, Amin MA. Bat diversity and abundance as indicators of disturbance in Neotropical rainforests. Conservation biology. 2000;14(6):1666-75.
42. Schulze MD, Seavy NE, Whitacre DF. A Comparison of the Phyllostomid Bat Assemblages in Undisturbed Neotropical Forest and in Forest Fragments of a Slash- and-Burn Farming Mosaic in Petén, Guatemala 1. Biotropica. 2000;32(1):174-84.
43. García-García JL, Santos-Moreno A, Kraker-Castañeda C. Ecological traits of phyllostomid bats associated with sensitivity to tropical forest fragmentation in Los Chimalapas, Mexico. Tropical Conservation Science. 2014;7(3):457-74.
44. Fernández-Montes de Oca AIF-M, Gallardo-Cruz JA, Ghilardi A, Kauffer E, Solórzano JV, Sánchez-Cordero V. An integrated framework for harmonizing definitions of deforestation. Environmental Science & Policy. 2021;115:71-8.
45. Williams-Guillén K, Perfecto I. Ensemble composition and activity levels of insectivorous bats in response to management intensification in coffee agroforestry systems. PLoS One. 2011;6(1):e16502.
46. Baker LE. Corn meets maize: food movements and markets in Mexico: Rowman & Littlefield Publishers; 2012.
47. Esteva G, Marielle C. Sin maíz no hay país2003.
48. Aguilar-Jiménez CE, Tolón-Becerra A, Lastra-Bravo X. Evaluación integrada de la sostenibilidad ambiental, económica y social del cultivo de maíz en Chiapas, México. Revista de la Facultad de Ciencias Agrarias Universidad Nacional de Cuyo. 2011;43(1):155-74.
49. SIAP. Cierre de la producción agrícola. In: Pesquera SdIAy, editor. Ciudad de México2023.
50. Wohlgemuth NH-M. Migration and indigenous communities in the southern states of Oaxaca and Chiapas, Mexico. Perspectives on Global Development and Technology. 2014;13(3):379-400.

51. Fernandez-Sanchez H. Transnational migration and Mexican women who remain behind: An intersectional approach. Plos one. 2020;15(9):e0238525.
52. Pérez PPR, Vázquez MRP, Daumás SH, Hernández OBH, Toral JN. Estrategias de vida, sistemas agrícolas e innovación en el municipio de Oxchuc, Chiapas. Revista de Geografía Agrícola. 2009;(42):83-106.
53. Cuero JM. Desagrarización del campo chiapaneco. Encrucijadas: Revista Crítica de Ciencias Sociales. 2011;(1):106-28.
54. Hilario FG, Morales JC, Ramírez AEC, Hamilton TT, Flores CP. Crisis del sistema milpero: la erosión biológica y cultural en San Juan de las Nieves, Malinaltepec, Guerrero, México. Revista de Geografía Agrícola. 2016;(57):113-23.
55. Solís JDÁ, Martínez MdJA. Actividad microbiana del suelo bajo diferentes sistemas de producción de maíz en los altos de Chiapas, México. Agrociencia. 2004;38(1):13-22.
56. Santiago-Lastra JA, Perales-Rivera HR. Producción campesina con alto uso de insumos industriales: el cultivo de repollo (brassica oleracea var. capitata) en los altos de Chiapas. Ra Ximhai. 2007;3(2):481-507.
57. Mariaca Méndez R, Jácome G, Martínez L. El huerto familiar en Méxicoavances y propuestas. Avances en agroecología y ambiente Vol 1/JF López Olguín, A Aragón García y AM Tapia Rojas (Eds) 2007p 119-138. 2007.
58. Escobar-Castillejos D, Caballero-Roque A, Osten R-V. Prácticas de utilización para plaguicidas en la localidad Nueva Libertad, La Concordia, Chiapas. Revista mexicana de ciencias agrícolas. 2011;2(SPE1):19-30.
59. von Osten JR, Garro DH. Uso de plaguicidas altamente peligrosos en Campeche. Los Plaguicidas Altamente Peligrosos en México. 2015:309.
60. Bernardino Hernández HU, Mariaca Méndez R, Nazar Beutelspacher A, Álvarez Solís JD, Torres Dosal A, Herrera Portugal C. Percepciones del uso de plaguicidas entre productores de tres sistemas agrícolas en Los Altos de Chiapas, México. Acta universitaria. 2017;27(4):19-34.
61. Bernardino-Hernández HU, Mariaca-Méndez R, Nazar-Beutelspacher A, Álvarez-Solís JD, Torres-Dosal A, Herrera-Portugal C. Conocimientos, conductas y síntomas de intoxicación aguda por plaguicidas entre productores de tres sistemas de producción agrícolas en los altos de Chiapas, México. Revista internacional de contaminación ambiental. 2019;35(1):7-23.
62. Estrada A, Coates-Estrada R. Bats in continuous forest, forest fragments and in an agricultural mosaic habitat-island at Los Tuxtlas, Mexico. Biological conservation. 2002;103(2):237-45.
63. Racero-Casarrubia JA, Correa JB, Marrugo-Negrete J, Pinedo-Hernández JJ. Plaguicidas organoclorados en murciélagos (Chiroptera) asociados al bosque húmedo tropical en Córdoba, Colombia. Caldasia. 2021;43(2):320-30.
64. Torquetti CG, Guimarães ATB, Soto-Blanco B. Exposure to pesticides in bats. Science of the Total Environment. 2021;755:142509.
65. Bayat S, Geiser F, Kristiansen P, Wilson SC. Organic contaminants in bats: trends and new issues. Environment international. 2014;63:40-52.
66. Gándara G, Correa AN, Hernández CA. Ecological Service Offered by Tadarida brasiliensis Bats as Natural Plague Controllers in Northern Mexico and Their Economic Valuation. OALib. 2023;10(11):1-16.

67. Zapata-Morán I, Lopez-Feldman A, Bejarano HD. Tequila, bats, and agave farmers: Towards an understanding of the right incentives to protect genetic diversity. Environmental Research Communications. 2024.
68. Abson DJ, Termansen M. Valuing ecosystem services in terms of ecological risks and returns. Conservation Biology. 2011;25(2):250-8.
69. Blanco CA, Pellegaud JG, Nava-Camberos U, Lugo-Barrera D, Vega-Aquino P, Coello J, et al. Maize pests in Mexico and challenges for the adoption of integrated pest management programs. Journal of Integrated Pest Management. 2014;5(4):E1-E9.
70. Salinas JCC, Vidal HA, Cabañas AAG, Ramos EN, Cruz FA, Salinas EO. Morphological composition and yields of native maize without the use of agrochemicals in Chiapas, Mexico. Siembra. 2023;10(2).
