# Supplementary material for: Impact of climate change on the distribution of insectivorous bats: Implications for small-scale farming in southern Mexico
Source: PLoS One. 2024 Dec 2;19(12):e0310623. doi: 10.1371/journal.pone.0310623 (PMC11611147; doi:10.1371/journal.pone.0310623)
Supplement: Attachment — Submitted filename: RebuttalLetter.docx [file pone.0310623.s005.docx]

1. Reviewer #2: I reviewed the first iteration of this manuscript and applaud the authors for their great work in addressing the reviewers’ critiques and suggestions. I previously noted major concerns about the format of the questions and responses in the participant study – and in particular the conclusions that the authors drew from them. Now, the interpretations are much more careful, intriguing, and I feel they can galvanize conservation efforts in the area. I really liked that the authors’ argued for the validity of the farmers’ perceived decline in bats and that if these long-standing farmers are recognizing fewer bats, then we need to start monitoring and collecting data in the area immediately. I was also enlightened by the added cultural background and local context. Dropping the richness estimates improved the relevance of the SDM. The additional supplementary data was also very helpful and allowed me to better understand the study’s findings. As a result, I feel the spirit and substance of the manuscript is now integrated as per the authors’ original intent.

   I still feel there are still some areas that can improve the interpretability of the manuscript.

Answer: *Thank you for your time and feedback that helped improve our manuscript*.

2. Table 1 caption: While much improved from the original manuscript, the caption must be able to stand alone from the main text. First, in the caption, re-explain what the GCMs indicate (as per lines 135-141). Clarify that these values represent percentages of pixel loss/gain relative to contemporary projections. Re-clarify the spatial grain: what each pixel represents. Making these changes will help your readers immensely.

*Answer: We tried to clarify the Table caption to make it stand alone. We highlighted the Table caption in order to facilitate the view of corrections.*

Table 1 format: I don’t believe we need both the mean and median. Pick one measure of centrality that most honestly fits with the distribution of the data. The other measure will still be in the supplementary data. If the data are not a normal distribution, use the median (and vice versa). The current table’s `Range change` column is redundant, containing values that are already in the same row so it can be removed. If the authors’ still want to direct the readers to minimum and maximum values, the values in each row can be bolded or italicized (and specified in the caption).

Answer: *Done.*

Finally, I liked the format of the table in SupplementaryMaterial_II in the `Total`tab better. It is much more transparent with pixel information so I recommend using the tabular format of one of those tables. The authors must indicate that the `%Loss` column reflects the non-dispersal scenario and `Range change%`column reflects the limited-full dispersal column. The reason suppmat format works is because GCMs are the rows and the descriptive statistics are the columns.

Alternatively, consider pivoting the years for each GCM into descriptive statistic columns, if possible. That way the reader reads time from left to right. Still pick either the median or mean, depending on the distribution of the data.

Answer: *We changed the table as suggested. We hope it is clearer now.*

Lines 333 – 335: I recommend that the authors also include dietary monitoring as a conservation need, given the dearth of dietary information for species in the area and relevance to the study’s topic. DNA metabarcoding has made dietary screening more accessible than ever before. If bats are ever mist-netted, it is no issue to collect guano and send for DNA screening, so long as funding proposals include this as a goal. There are papers that have even used the DNA metabarcoding to demonstrate pest consumption as an ecosystem service in areas with small-scale agriculture and I don’t think it will be difficult for the authors to find a few either in their surrounding regions or others, like African nations, for example. Metabarcoding not only allows for pest detection but allows acquisition of dietary diversity so that changes in consumption patterns can be monitored. Perhaps, dietary data could also clarify other perceptions of the farmers (e.g., are the bats also eating their corn crop?).

Answer: *Thank you for your comment. It is a great goal and would be amazing to achieve it. We incorporated this information in our text and added the following reference:*

Gonçalves, A., Nóbrega, E. K., Rebelo, H., Mata, V. A., & Rocha, R. (2024). A metabarcoding assessment of the diet of the insectivorous bats of Madeira Island, Macaronesia. *Journal of Mammalogy*, *105*(3), 524-533.

***It would be wonderful to think in a future collaboration if you might be interested.***

Minor comments:

Line 46: “Surveyed farmers perceived a decline in bat populations” – percentage needed.

Answer: *Done*

Lines 68-69: This sentence is out of place. I would delete it or move and integrate into the final paragraph of the introduction.

Answer: *We deleted it.*

Lines 75 – 77: Consider starting a new paragraph with this sentence because it functions as a topic sentence for the remaining sentences on the local society and culture.

Answer: *Done*.

Lines 80 – 87: These sentences should be moved and integrated into the final paragraph of the introduction.

Answer: *We rephrased following your advice.*

Line 102: delete “there”

Answer: *Done.*

Line 105: replace comma with semicolon.

Answer: *Done.*

Line 318: $16’899,132 – replace apostrophe with comma

Answer: *Done*.

Line 325: Delete “do”

Answer: *Done*.

Lines 327 – 328: Sentence is a bit wordy and difficult for me to follow. Consider the following line correction: “Insectivorous bat distributions under both climate scenarios will likely have negative impacts …” Or determine another revision for readability.

Answer: *Done*.

Lines 349 - 351: I do not understand this sentence. Revise for clarity.

Answer: *Done*.
